# 135. Comparing the Bioinformatic and Experimental Approaches for Identifying Antibiotic Resistance Genes on Plasmids of *Acinetobacter baumannii*

**DOI:** 10.1093/ofid/ofac492.213

**Published:** 2022-12-15

**Authors:** Munok Hwang, Hosoon Choi, Chetan Jinadatha, Jing Xu, Thanuri Navarathna, Landon Ashby, Morgan Bennett, Keith S Kaye, Sorabh Dhar, Piyali Chatterjee

**Affiliations:** Central Texas Veterans Health Care System, Temple, Texas; Central Texas Veterans Health Care System, Temple, Texas; Central Texas Veterans Health Care System, Temple, Texas; Central Texas Veteran Health Care System, Temple, Texas; Central Texas Veterans Health Care System, Temple, Texas; Central Texas Veterans Health Care System, Temple, Texas; Central Texas Veterans Health Care System, Temple, Texas; Rutgers - Robert Wood Johnson Medical School, New Brunswick, New Jersey; Wayne State University/Wayne Health, John Dingell VAMC, Detroit, Michigan; Central Texas Veterans Health Care System, Temple, Texas

## Abstract

**Background:**

Antimicrobial resistance (AMR) genes of bacteria can be found in chromosomes or plasmids. Due to horizontal transfer from one bacterium to another, AMR genes on plasmids can be easily spread. However, distinguishing whether AMR genes are from plasmids or chromosomes is difficult. Here, we assessed two different approaches for identifying AMR genes on the plasmids of *A*. *baumannii*.

**Table 1.**
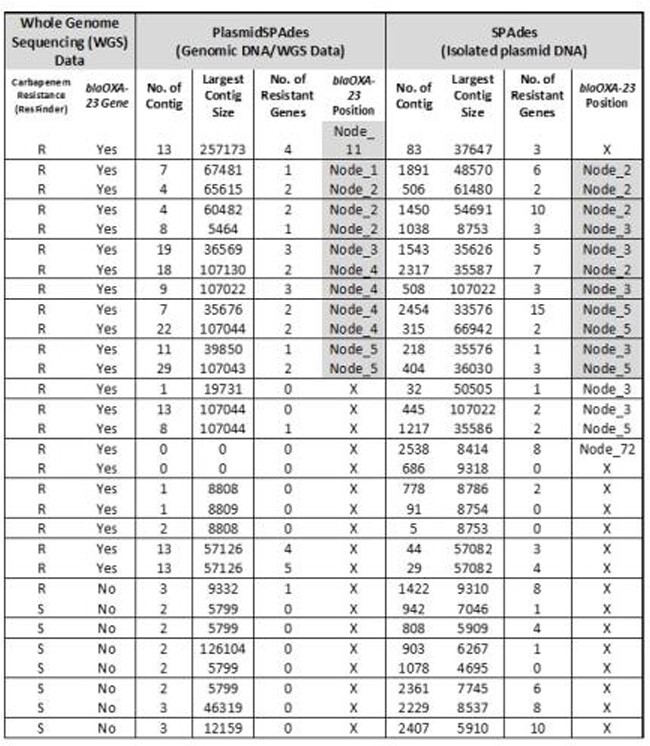
Comparison of informatics and experimental approach in studying AMR genes on plasmids in A. baumannii

**Methods:**

Genomic DNA from 30 clinical isolates was sequenced using Illumina NextSeq 550. Two approaches were applied to identify which AMR genes were on plasmids: isolation of plasmid DNA experimentally using plasmid isolation kit (Qiagen, CA), and deduction of plasmid sequence from genomic DNA using the bioinformatic tool, plasmidSPAdes, which distinguishes the plasmid sequence based on the copy number. AMR genes from both approaches were identified using the open-source AMR database, ResFinder. The AMR gene data obtained from plasmids utilizing each approach were compared based on information from whole genome sequencing (WGS) data.

**Results:**

As shown in Table 1, both approaches produced differences in the number of contigs, largest contig size, and number of AMR genes identified. The average number of contigs using plasmidSPAdes with WGS data was 7.4 and the largest contig size was 52939 base pairs, while with isolated plasmid DNA and analyzed by SPAdes was 1025 and 32274 bp, respectively. Plasmid DNA contigs are more fragmented. The average number of AMR genes identified using plasmidSPAdes was 1.2 and using plasmid DNA was 4. AMR information from WGS data showed 73.3% isolates have *blaOXA-23,* a carbapenem resistant gene. However, *blaOXA-23* was found from 40% isolates using plasmidSpades and 50% isolates using plasmid DNA. In 36.7% isolates, *blaOXA-23* was found by both approaches.

**Conclusion:**

The information obtained by the two approaches for identifying resistance genes on plasmids mostly agreed with some exceptions. The *bla OXA-23* identified in both could be on plasmids, while genes not identified from both could be on chromosomes. PlasmidSPAdes was a useful tool to discriminate plasmid sequence from WGS data. Stringent infection control measures can prevent spread of AMR gene containing plasmids in hospital settings.

**Disclosures:**

**Chetan Jinadatha, MD, MPH**, AHRQ R01 Grant-5R01HS025598: Grant/Research Support|EOS Surfaces: Copper Coupons and materials for testing **Keith S. Kaye, MD, MPH**, Allecra: Advisor/Consultant|GlaxoSmithKline plc.: Receiving symposia honoraria|GlaxoSmithKline plc.: GlaxoSmithKline plc.-sponsored study 212502|Merck: Advisor/Consultant|qpex: Advisor/Consultant|Shionogi: Grant/Research Support|Spero: Advisor/Consultant **Piyali Chatterjee, PhD**, AHRQ Grant # 1R03HS027667-01: Grant/Research Support|AHRQ Grant # 1R03HS027667-01: Central Texas Veterans Health Care System.

**Table 1.** Comparison of informatics and experimental approach in studying AMR genes on plasmids in A. baumannii

*Highlights indicate blaOXA-23 genes found in both approaches.

*S-Susceptible isolates with no blaOXA-23 gene serve as control

